# Rheumatoid arthritis patients' experiences of wearing therapeutic footwear - A qualitative investigation

**DOI:** 10.1186/1471-2474-8-104

**Published:** 2007-11-01

**Authors:** Anita E Williams, Christopher J Nester, Michael I Ravey

**Affiliations:** 1 Directorate of Podiatry, University of Salford, Frederick Road, Salford, UK; 2 Centre for Rehabilitation and Human Performance Research, Brian Blatchford Building, University of Salford, Salford, UK; 3 School of Nursing, Allerton Building, University of Salford, Frederick Road, Salford, UK

## Abstract

**Background:**

Specialist 'therapeutic' footwear is recommended for patients with diseases such as rheumatoid arthritis (RA) as a beneficial intervention for reducing foot pain, improving foot health, and increasing general mobility. However, many patients choose not to wear this footwear. Recommendations from previous studies have been implemented but have had little impact in improving this situation. The aim of this study was to explore RA patients' experiences of this footwear to ascertain the factors which influence their choice to wear it or not.

**Method:**

Ten females and three males with RA and experience of wearing specialist footwear were recruited from four National Health Service orthotic services. Semi-structured interviews were carried out in the participants own homes. A hermeneutic phenomenological analysis of the transcripts was carried out to identify themes.

**Results:**

The analysis revealed two main themes from both the female and male groups. These were the participants' feelings about their footwear and their experiences of the practitioner/s involved in providing the footwear. In addition, further themes were revealed from the female participants. These were feelings about their feet, behaviour associated with the footwear, and their feelings about what would have improved their experience.

**Conclusion:**

Unlike any other intervention specialist therapeutic footwear replaces something that is normally worn and is part of an individual's body image. It has much more of a negative impact on the female patients' emotions and activities than previously acknowledged and this influences their behaviour with it. The patients' consultations with the referring and dispensing practitioners are pivotal moments within the patient/practitioner relationship that have the potential to influence whether patients choose to wear the footwear or not.

## Background

In the UK at least 7 million people are disabled and it is estimated that 400,000 of these will require aids in the form of specialist 'therapeutic' footwear, orthotic devices, callipers and footwear adaptations [1]. The National Health Service expenditure associated with this is £80 million per year [1], with an estimated 25% of the budget being spent on this footwear, which is provided to patients with a variety of pathologies associated with systemic diseases such as rheumatoid arthritis (RA).

Rheumatoid arthritis has significant effects on foot structure [2,3] with an estimated prevalence of structural and related problems of up to 50% [2], increasing to 90% in the older person with RA [4]. There is some evidence that therapeutic footwear is a beneficial intervention [5,6] in achieving reduced pain, improved foot health, and increased patient mobility. Further to this evidence, the provision of this footwear is supported by national guidelines [7]. However, the most significant barrier to achieving these benefits is patients choosing not to wear the footwear. Patient dissatisfaction and variable levels of usage have been identified in several studies [8-11] and every report [1,12,13] that has sought to investigate footwear use, with the fate of this footwear being described as;

"...shoes in the cupboard" [11].

Therefore, this footwear may not be the panacea for foot problems that the practitioners prescribing it presume it to be and the expected foot health improvements are negated by the fact that patients choose not to wear it.

The reasons why patients make the choice not to wear the footwear are complex in relation to the footwear itself, the service which provides it and the influences that affect the patient before and after receiving it. The majority of most previous research has focused on patient dissatisfaction with the appearance of the footwear and the lack of compliance with the practitioners' instructions to wear the footwear [8-10].

Previous research on this topic has employed questionnaires to ascertain the perspectives of patients. However, these questionnaires are generally focussed on the problem areas as viewed by the practitioner [8-10].

Services that provide the footwear have been evaluated to a limited extent. Although some evidence supports the perspective that a multidisciplinary approach may have a positive influence on patient compliance, its impact is limited [11,14]. Patients with chronic diseases such as RA have complex needs and therefore the factors that influence their choice to wear the footwear are potentially complex. These factors may not have been identified in previous studies, evidenced by the fact that there is still the problem of patients not wearing this footwear. Therefore, the aim of this study was to explore RA patients' experiences of being provided with specialist footwear and to identify factors, which may contribute to their choice to wear it or not.

## Method

An interpretive phenomenological approach [15] was adopted and from this philosophical stance semi-structured interviews were used to explore the participants' personal experiences of using therapeutic footwear. The interviews and the analysis of the data were carried out by a researcher (AW) with previous experience of this area from both a clinical and research context. Self-reflection on this previous experience took place and continued through the interpretative process. This relates to the process by which the new meaning that has been revealed from the data is fused with the previous understanding and new knowledge or understanding is produced [16].

Following approval from a local ethical committee, participants were recruited from orthotic services in four hospitals within the North West of England. The following inclusion criteria were applied in relation to participant selection:

• Listed on orthotic service records

• Attended a clinic appointment for footwear within the last six months

• Received specialist footwear from the orthotic services

• Reported at their last appointment that they were satisfied with the footwear

• Had a diagnosis of rheumatoid arthritis [17]

Fourteen people meeting these criteria were sent letters and information leaflets about the planned study and out of these ten women and three men agreed to take part in the study. One patient declined to take part due to an impending planned hospital admission.

All interviews were carried out in the participant's own home and there were no third parties present during the interview. Following an introduction by the researcher (AW), further clarification of the purpose of the study was given and an opportunity for the participants to ask questions was provided. Informal conversation took place before the interview in order to put the participants at their ease. Formal written consent was obtained and all participants agreed to proceed to the interview. They were assured that they could stop the interview at any time and withdraw from the study if they so wished. The participant's gender, age, duration of RA disease, level of foot pain (visual analogue scale) and current footwear usage was documented. The interviews were recorded on a digital voice recorder. The researcher (AW) asked an initial question, "*Tell me about your experiences of being provided with and wearing specialist footwear"*. Dependent on the participants' responses, further trigger questions were used if the conversation came to a halt or if the participant wandered off the subject of footwear for too long a period of time.

The additional questions included:

• *"How did you feel when you first wore the footwear?"*

• *"How do you feel about it now?"*

• *"What impact has the footwear had on your ability to do the things you want to do?"*

• *"Are there any aspects of the footwear that you would like to comment on?"*

• *"Are there any aspects of the service which you would like to comment on?"*

The interviews were transcribed verbatim immediately after the interviews by the researcher (AW) and pseudonyms were used to replace the participants' names to ensure confidentiality. The transcripts were then returned to the participants for confirmation of the content.

Analysis was carried out after transcription of all the interviews using a hermeneutical approach [16,18]. Each transcript was read several times and significant statements and meanings were identified. These statements and meanings were then organised into main themes which formed the basis of the results. The transcripts were then analysed by a second researcher to add to the reliability of the initial analysis. Exemplars from the transcripts were identified to support each of the themes. This is a presentation strategy that allows the reader to visualise the person in the situation. By showing examples of the phenomena to the reader this allows further interpretation and understanding of the text and the true 'authentic' nature of the experiences to be revealed. The provision of these examples also supports the trustworthiness of the findings [18]. To support the richness of the data and to add to the trustworthiness of the data a reflective journal was kept by the researcher. The journal was used within the analytical process to support how the participants responded both verbally and non-verbally in addition to what they revealed in open dialogue.

## Results

All the participants are described in terms of age, duration of disease, foot pain and current footwear usage (Table 1).

**Table 1:** Participant demographics

	FEMALES	MALES
Age (yrs)	Mean 59 range 44–76	Mean 53 range 50–57
Disease duration (yrs)	Mean 14 range 5–26	Mean 6 range 4–12
Foot pain (Likert score)	Mean 8 range 7–10	Mean 7 range 7-7
Footwear usage	8 currently used ; 2 not used	3 currently used

Significant statements and meanings emerged from analysis of the transcripts that were identified as factors, which contribute to their health behaviour and choice to wear or not to wear the footwear. These statements were then organised into themes. The analysis revealed two main themes from both the female and male groups. These were the participants' feelings about their footwear and their feelings and experiences of the practitioner/s involved in providing the footwear. Additional themes were revealed from the female participants, which were feelings about their feet, behaviour associated with the footwear, and their feelings about what would have improved their experience.

### Theme 1 – Feelings about their feet (female group)

The female participants expressed feelings about how their feet were visibly different from other people who they considered 'normal'. This visibility was identified as being how the feet looked and how they impacted on their ability to walk 'normally' because of the pain. None of the male participants talked about the appearance of their feet. The females had concern for how others see them, the loss of femininity and demonstrated emotions such as frustration, anger, anxiety, loss and sadness, which were expressed both verbally and in the way these statements were said.

".....well it shows in your face...the pain you know...maybe because you are stood on them...makes you self conscious as well...I look and feel like an old lady". Rose

"I can't walk normally... If you shuffle around people notice and (thinks for a time)........it shows in your face...pain shows in your face...makes you look old and I feel ...when I look in the mirror god what must my husband think..." Lily

"I feel and look rubbish most of the time.... I don't feel feminine or sexy any more...especially how my feet look...they look awful...... ". Daisy

### Theme 2 – Feelings about their footwear (Female and male group)

Statements and meanings about the footwear itself formed the second theme. In the female group, the negative feelings and emotions about their feet were reinforced by the reaction of 'others' to the footwear. They considered themselves as being visibly different whilst wearing the footwear and this impacted on how they viewed themselves and how they viewed that others saw them. Again, the emotions of shame, sadness, and anger were not only identified as being associated with their feet but also with the footwear.

"The shoes....as soon as I see a person I can say oh yes she's got hospital shoes on..... I compare my boots with other people and they are more feminine and pretty and that makes me feel sad". Daphne

"I think I knew that the shoes had to... well...like...treat my feet...its like wearing a splint....I have some of those for my hands...but shoes are different...they are meant to make you look nice...in my early 20s I used to wear sexy stilettos...no chance now even if I don't walk in them (looked sad)". Carol

"I felt very tearful the first time I had the shoes...humiliated....I would get dressed up and then I feel like a clown... just don't feel dressed...I feel untidy...that makes me angry". Lena

Just one of the male participants made a comment about the visible impact of the footwear and this was in relation to walking speed,

"I can walk faster in these shoes, which is good so I don't hold people up.." Arthur

This response is different than the females, who talked about the visibility of the footwear as an item of clothing. The main focus of the male participants was the construction of the footwear and the fact that it was free.

"I am really pleased with these shoes ...to have a pair of hand made shoes makes you feel great..." Arthur

"... oh this footwear is great...good and strong and my toes are comfy now caus they have soft linings in them ...they're hand made you know." George

### Theme 3 – Behaviour with the footwear (female group)

The loss of femininity and how the footwear impacts on the female participants' sexuality was further expressed through how it affects the participants' behaviour. This impact on their behaviour with the footwear became the third theme. This theme was not apparent in the men's dialogue, with no mention of changed behaviour. The female participants, who had chosen to wear the footwear, did acknowledge that it improved their mobility in respect of reducing foot pain. However, it did restrict activities, particularly those perceived as social ones. There was evidence of compromise, with the footwear influencing the types of clothes worn. Trousers were viewed as being more suitable to wear with the footwear rather than dresses.

"I can't complain as I feel my mobility would not be as good with out these...Its important to be mobile and pain free...on a day to day basis they are just about ok...but when I go out I wear other shoes then pay for it after." Rose

"....I don't go out much...I can't get normal shoes on ...I am stuck with these..I can go shopping but I don't go to family do's...that makes me sad". Joan

"Well I suppose that I have a pair of shoes I can wear when things get really bad and at least I have something to put on my feet if I get really desperate... they do restrict what you can wear though, I do tend to wear trousers a lot and I would love to wear a feminine dress..". Maud

"Don't go out much but sometime makes me feel like crying and I panic when I do get an invite 'caus I think oh gosh these boots". Lena

Again the emotions of shame, sadness, and anger were identified as being associated with the impact of this footwear on the females participants' activities in addition to the feelings about their feet and the footwear.

### Theme 4 – Feelings about the practitioner (Female and male groups)

Statements and meaning in relation to the participants' feelings about the dispensing practitioner formed the fourth theme. It was expressed that they had trust in the practitioners' skills in the assessment and dispensing of the footwear. Two female participants described the practitioner as being a 'nice person', which was linked to them visibly trying options if there were issues or problems with the fit of the footwear. However, there were several negative attributes identified by the remaining female participants, for example, being dismissive of the patients concerns and having poor communication skills.

"There was no discussion....if fact I don't really think it mattered what I thought ...just said I had difficult feet and that made me feel ashamed". Daisy

"I don't feel there was a two way conversation about what was available and how much say I had in what was going on ...". Daphne

The female participants perceived that the practitioners lacked knowledge about RA, pain and their needs in relation to footwear.

"I think generally there is not much understanding about how rheumatoid arthritis affects the person...well...we are people aren't we ....we are just not a pair of feet we have feeling... and sometimes I feel that they (practitioners) don't understand....". Alison

The recurring expression of the emotions of shame, sadness, and anger were expressed by the females as being associated with the consultation. Additional emotions of guilt and feelings of powerless were also expressed.

"I had chosen two pairs that I liked and after he had measured me he said I couldn't have those and pointed out a pair in the catalogue ... I asked if I could perhaps try the other and he said there was no point as my feet were really bad... made me feel guilty and ashamed". Joan

"I don't need...how can I say? (thinks) sympathy is wrong...it makes me feel...well its not helpful...condescending...its not a positive thing at all...makes me feel pathetic and powerless and now it makes me angry". Yvonne

The nuances of the non-verbal aspects of the therapeutic relationship were perceived as important by the women involved, with the practitioners' body language, such as head scratching or looking concerned, being mentioned as reinforcing these feelings.

"He pulled a face when he saw my feet....that made me feel... ashamed really..... and upset". Lily

The female participants' perception of the practitioners' attitude tended to reinforce the negative view of their foot problems and issues in relation to the footwear. In contrast, there was an air of camaraderie between the male participants and the male practitioners. All the men expressed trust in the skill of the practitioners and described them as 'friendly' and 'nice chaps'.

"The chap that makes them is good at his job...supports Man U (football team) too so we get on great!" John

The male participants did not mention the requirement for the practitioners to have knowledge of their condition. The only negative comment was that two of the participants expressed concern that they got to know one practitioner and then they moved to another hospital. This reflects the known problems with continuity inherent in the contracting arrangements for these practitioners with the National Health Service.

### Theme 5 – Feelings about what would have improved their experience (female group)

The female participants were very clear about what would have improved their experience and the statements in relation to this formed the fifth theme. The opportunity for time to consider their options before being referred for the footwear, to have more information on which to base their choices and to be able to voice their opinions were identified by the female participants as factors that would have improved their experience.

" I would have liked more choice as to whether to have the footwear in the first place... I felt I didn't have time to consider whether I wanted it or not....just....well...went along with what the doctor said". Daisy

In addition, knowing that they were being listened to and the feeling of trust in the practitioner were seen as important factors in the consultation.

"It is good to talk to someone who listens even though there is nothing that can be done...if you understand it helps .....trouble shared I suppose and all that....I feel very sad that I wasn't listened to...if I had it might have been better." Maud

"I think that the fitter....well any health person needs to listen to us more...I don't feel I was listened to about the footwear and now I feel guilty that I don't wear them...what a waste...that makes me angry." Yvonne

"Communication is a big word it is a very...you know knowing what's available...that alone ...if I had known what was going on yeah definitely knowing what is available and a two way thing..." Alison

"you assume they know their job but we know our bodies don't we ..I know what will work....and its not just a matter about what will work for our bodies ...it has to feel right.....look right and ...well its more about how we feel in the head isn't it?.." Carol

In contrast the male participants did not mention any aspect of their experience that needed improving. Acknowledgement by the practitioner that the females had unique knowledge of their own disease would have made them feel important and included in the process, would have enhanced their experience and perhaps avoided some of the negative emotions.

## Discussion

The depth of information obtained from these participants has revealed some extremely important issues in addition to what is known from the existing literature that have used more quantitative methods such as questionnaires [8-10]. The interviews have revealed a richness of data about the participants' feelings, perceptions and behaviour with the footwear, particularly in the female group. The strength of this research lies in the fact that the participants who were recruited for this study were considered satisfied by the service that provided the footwear. If the participants were deemed dissatisfied with their footwear at recruitment it could be said that the results were biased.

Despite being thought of as being 'satisfied' the females in this study revealed aspects with which they were not satisfied but they demonstrated gratitude for the practitioners' interventions. This appreciation may be what is reported in previous questionnaires as satisfaction [8-10]. This implies that they are not entirely satisfied but feel that they should be grateful for anything even if it is not satisfying all the aspects associated with footwear use.

That the female participants initially focussed on their feet as an issue, highlights the inseparable combination of feet *and *footwear, with the footwear reinforcing their already negative feelings about their self image. This negative self image is known to exist in this patient group [19] and in this situation could be compounded by this footwear. This footwear is required to meet their needs as defined in the retail context in that footwear is chosen and is worn as an item of clothing. This is quite different to the specialist 'therapeutic' footwear which is supplied by the NHS as an intervention to 'use' rather than an item that is 'worn'. This being the case, the usual choice of which footwear to wear is automatically being taken away from the patient since their existing choice of retail footwear has been deemed unsuitable for their foot health needs.

The replacement of their existing footwear is often without the patient's pre-emptive request or involvement in the decision [11]. From these findings it can be interpreted that the patient has far less control and influence in the choice of specialist footwear compared to their usual retail purchase. This is likely to disengage the patient because we are telling them their previous choice of footwear was wrong, and we know what is best for them, which by default confines the patient to the role of passive recipient rather than the desired active participant. Often the only choice the patient has is whether to wear the specialist footwear once it has been supplied. The fact that evidence [8-11] suggests that patients frequently choose not to wear this footwear after it is supplied indicates that in the context of the consultation, the practitioner has failed to establish the patient as an active participant in the decision making before the footwear is supplied.

In the female group, the footwear itself and its impact on restricting their choice in clothes could be seen as a contributing factor to social isolation which is evident in people with chronic disease [20,21]. The male participants found the visual appearance of their footwear acceptable as it is more suitable for use with trousers and therefore loss of choice was not an issue.

In this study, two female participants chose not to wear the footwear at all and had relegated the footwear to 'shoes in the cupboard' [11]. This reflects the results of previous work that identified that one in six pairs of shoes are not worn [12]. This choice was not taken lightly and it was evident that this action invoked feelings of anger because their expectations had not been met. Despite the footwear being free, all female participants recognised a cost to the NHS and therefore when they chose not to wear it they felt guilty at the waste.

The focus of much of the female participants' dialogue was around the consultation with the dispensing practitioner, the majority of which were negative aspects. The body language of the practitioner and their attitude reinforced both the female participants' negative feelings and guilt at being perceived as a 'difficult' patient. This possibly reinforces the balance of power with the dispensing practitioner and would not encourage open and shared dialogue. In some cases the participants perceived the practitioner as using guilt about the cost of the footwear to encourage its use. It is not clear whether the cost of the footwear is a legitimate area to discuss as it places guilt as the primary motivation to wear them rather than the patient choosing to wear them. Guilt is a negative emotion and again places the balance of power with the dispensing practitioner. The participants expressed that they perceived the dispensing practitioner as having little or no knowledge of RA and the impact of pain. Whether these practitioners did have knowledge of RA is not known but the importance is that they did not demonstrate this to the patient. In addition, the lack of opportunity for the participants to have a voice detracts from the development of a therapeutic relationship. This problem was augmented by the lack of continuity of care by the dispensing practitioners due to the service contracting arrangements whereby the practitioner may be different at each patient visit.

The female participants demonstrated knowledge about their foot problems and had opinions as to what would have helped if they had been listened to. The possession of this knowledge identifies them as experts of their own problems, which in turn leads to the expectation that they would have strong control and a sense of ownership in the decision making process. The imbalance of the existing process supports their role as passive recipients with no control and therefore it is not surprising that control is only regained in the choice of wearing or not wearing the footwear once it has been dispensed. There was evidence of trust in the dispensing practitioner to know their job with reference to the footwear itself and they were perceived as the experts in this footwear. However, the female participants expressed that they were the experts in their condition and in their own unique experience of it. That patients are experts in their own condition is recognised specifically in patients with RA but in conjunction with this they need information and guidance that enables them to make informed choices of service providers and the treatments they offer [7].

The consultation between the participants and the dispensing practitioner was identified as a vital component affecting whether the patients engage in the use of specialist footwear. The relationship between the patient and the practitioner is seen as a partnership in which information, feedback on progress, support and empathy by the practitioner is necessary to influence the patient's perceptions of control in a positive way [22]. It is already known that the clinical encounter with the practitioner is perhaps the most important factor in the patient's engagement with health interventions [23]. Street [24] identifies that;

"... *in spite of sophisticated technologies for medical diagnosis and treatment, talk remains the primary means by which the physician and patient exchange health information*".

To promote positive health behavior, the balance of power in the patient/practitioner relationship needs to shift towards the patient so that they are not a passive recipient but become an active participant in the decision making[25].Establishing a concordant relationship through effective communication facilitates the patients control over choices and their subsequent engagement in the intervention [25]. The nature and composition of the clinical consultation about specialist footwear is potentially a significant factor influencing whether the patient chooses to wear it or not. Most participants in this study behaved as passive recipients with apparently no opportunity to choose to reject the option of footwear before they are referred to the dispensing practitioner by the referring practitioner. Therefore, the source of the problem with specialist footwear could be perceived to be at the point of referral with the template for the balance of power set at this event to be with the practitioner. Knowledge about specialist footwear by the referring practitioner and engaging with the patient in the decision to refer is a pivotal moment at which the patient either becomes a passive recipient or an active participant using information to make considered choices.

### Recommendations for practice

Referring practitioners need have an understanding of how foot problems impact on the individual patient from the patient's perspective with regard to appearance as well as function, an understanding of the limitations of the specialist footwear and what options are available other than the footwear, such as foot surgery.

Effective consultation skills should be integrated into undergraduate and postgraduate training for all practitioners involved in the provision of specialist footwear. There is evidence to support the effectiveness of these consultation skills being taught in other areas [26,27].

## Conclusion

The approach adopted in this study has facilitated the participants to acquire a voice regarding their experiences of therapeutic footwear. In particular, this wealth of evidence demonstrates that therapeutic footwear, unlike any other intervention replaces something that is normally worn and is part of an individual's body image. To achieve the potential positive health benefits, this footwear has to fulfil both the clinical requirements and acknowledge the social expectations of women. The consultation with the referring practitioner and the dispensing practitioner are pivotal moments that have the potential to influence whether patients chose to wear the footwear or not.

When a more patient centred approach is adopted in all aspects of the referral and dispensing process we can then evaluate whether more patients chose to be referred for this footwear, and choose to wear the footwear.

## Key messages

• Specialist therapeutic footwear impacts more on the patients body image and emotions than previously acknowledged, particularly in respect of female patients and even in those who are considered as 'satisfied'.

• Being understood and being able to voice their opinions increase the patients feeling of being important and this 'importance' may positively impact on their choice to wear the footwear.

• Training of practitioners in a more patient focussed consultation style may improve the patients experience and engagement in the footwear as an intervention as well as something which is 'worn'.

## Competing interests

The author(s) declare that they have no competing interests.

## Authors' contributions

AEW conceived of the study and conducted the interviews and data analysis. MIR and CJN provided advice on the methodology and the procedural aspects of the study. MIR provided guidance during the analytical stage and helped with the manuscript. All authors read and approved the final document.

## Pre-publication history

The pre-publication history for this paper can be accessed here:


